# Prevalence of Postoperative Deep Vein Thrombosis in Patients Undergoing Emergency Laparotomy: A Prospective Observational Study

**DOI:** 10.7759/cureus.64932

**Published:** 2024-07-19

**Authors:** Preeti Acharya, Sudhir K Singh, Summi Karn, Udit Chauhan, Farhanul Huda, Somprakas Basu

**Affiliations:** 1 General Surgery, All India Institute of Medical Sciences, Rishikesh, Rishikesh, IND; 2 Radiology, All India Institute of Medical Sciences, Rishikesh, Rishikesh, IND

**Keywords:** exploratory laparotomy, postoperative complication, pulmonary embolism (pe), acute care surgery and trauma, deep venous thrombosis (dvt)

## Abstract

Background

Deep venous thrombosis (DVT) is more common in the hospital population and has an estimated annual incidence of 67 per 100,000. Surgery is a risk factor for DVT and has been proven to cause morbidity and mortality in the postoperative period. The correlation between the frequency of DVT and major surgical procedures has been demonstrated. However, few studies have been conducted on the relationship between emergency surgeries and the incidence of DVT. Our study aims to determine the prevalence of postoperative DVT in patients undergoing emergency laparotomies.

Methods

This prospective observational study was conducted over 18 months, from January 2021 to July 2022. Patients who underwent emergency exploratory laparotomies were included in this study. Duplex ultrasonography was done preoperatively to exclude patients with DVT. A serial duplex ultrasound was performed to detect DVT until the seventh postoperative day. All the clinicopathological and surgical information of patients relevant to this study was collected and analyzed.

Results

Out of 146 patients, one developed DVT in the postoperative period. The patient who experienced DVT had no other known risk factors; however, his age of 60 was a risk factor for DVT. So, the reported prevalence of DVT in our study population was just 0.68% of emergency exploratory laparotomy patients.

Conclusion

Our study reports DVT in only one case out of 146 patients who underwent emergency exploratory laparotomy without routine pharmacological prophylaxis. We might, therefore, conclude that emergency exploratory laparotomy may be a separate risk factor for the emergence of DVT. More prospective studies with large sample sizes should be done to evaluate the prevalence of DVT in emergency exploratory laparotomies.

## Introduction

Deep venous thrombosis (DVT) and pulmonary embolism (PE) are much more common within the hospital population due to various risk factors and have an estimated annual incidence of 67 per 100,000 among the general population [1-3]. Surgery is a well-established risk factor for the development of DVT [4]. Operative emergency general surgery (EGS) cases have been shown to have higher rates of DVT when compared with non-EGS cases [5]. A significant retrospective study in 2015 found that 15.6% of EGS patients experienced postoperative DVT requiring treatment. In contrast, only 9.1% of non-EGS patients similarly experienced postoperative DVT [2,5]. Morbidity can be reduced only through early diagnosis and treatment.

A significantly greater risk of DVT and PE is linked to all surgeries, particularly major orthopedic and neurovascular procedures, especially in patients with additional risk factors such as advanced age, prior DVTs, and underlying medical conditions. Thus, proven risk factors for DVT in the Indian population mainly include age, obesity, smoking, major surgery, trauma, prolonged bed rest/immobilization, pregnancy or puerperium, drugs (oral contraceptives, post-menstrual hormones), previous history of DVT, and some genetic factors (like antithrombin III, protein C and S deficiency) [6].

As we know, surgery is a well-established risk factor for developing DVT and thromboembolism, which have been proven to cause morbidity and mortality in the postoperative period. In the Western population, venous thromboembolism (VTE) prophylaxis is given after major surgeries and has been shown to reduce the risk of postoperative VTE [7]. However, there is no such protocol for initiating anticoagulants in the Indian population. The prevalence of DVT has remained constant for men and is rising for older women, while PE has decreased with time [6]. We must clearly understand how often DVT occurs in the Indian population and its impact on the health care system.

The Wells score is commonly used to predict VTE, whereas the D-dimer is frequently used for screening purposes. D-dimers have a high sensitivity but low specificity for detecting DVT or PE [8]. A complete duplex ultrasound is the imaging modality of choice for diagnosing acute and chronic DVT. While making the DVT diagnosis in our study, duplex ultrasonography was considered.

Whether elective or emergency, DVT and PE complications may occur after exploratory laparotomy [9]. Compared to non-EGS procedures, EGS entails a disproportionately high risk of postoperative complications, including VTE and mortality. Nevertheless, few studies have been conducted on the relationship between emergency surgeries and the incidence of DVT [10]. Western nations have invested enough in research to have DVT prophylaxis recommendations for both emergency procedures and all major abdominal surgeries. However, it turns out that most studies are retrospective.

Epidemiological information on the prevalence of VTE in Asian people is incredibly hazy and unclear. In Asia, VTE has traditionally been thought of as a rare disease. This notion, however, is debunked by fresh new findings. Due to disagreements over its incidence, the issue of routine VTE prophylaxis has generated significant debate throughout Asia [11]. While most research asserts that Asian patients experience DVT less frequently than their Western counterparts, new studies from this region have revealed a notable frequency of DVT in high-risk surgical patients [6,12,13]. However, in India, there are very few studies regarding the occurrence of DVT in emergency operative procedures. Additionally, there is little research in the Indian community to provide accurate instructions for initiating DVT prophylaxis. We need to know the burden of DVT in surgical emergency patients, as there is significantly less research on the occurrence of DVT in the Indian population. Our study aims to evaluate the prevalence of DVT in patients undergoing emergency exploratory laparotomies.

## Materials and methods

This prospective observational study was conducted in the Department of General Surgery, All India Institute of Medical Sciences, Rishikesh, Uttarakhand, India, after obtaining approval from the Institutional Ethics Committee of All India Institute of Medical Sciences, Rishikesh (approval number: AIIMS/IEC/21/120). A consecutive sample of patients who were undergoing emergency laparotomies was recruited over 18 months, from January 2021 to July 2022. Patients aged more than 18 years undergoing exploratory laparotomy and willing to participate in the study were included in this study. Patients with a previous history of DVT, intubated patients, patients on anticoagulant therapy, preoperatively diagnosed COVID-positive patients, and those who were diagnosed with DVT in the preoperative USG Doppler were excluded from the study.

For sample size, we used the Cochran equation (1997) N = Z2 PQ / e2, where N = total sample size, Z = 1.96 (at the 5% significance level), and P = prevalence of DVT taken from the previous study in India with a 5% margin of error (e). Thus, a minimum of 106 participants were planned to be included in this study.

A participant information sheet containing the details of the study was provided to the participants after obtaining valid informed consent. The demographic profile and risk factors associated with DVT were recorded. A preoperative USG Doppler and D-dimer were done, and the patients' APACHE II score was calculated. On postoperative days 3 and 7, a screening USG Doppler and D-dimer were performed, and the APACHE II score was also calculated.

A normal D-dimer was considered less than 0.50 (mg/L FEU), whereas a value of 0.50 or greater was considered a positive D-dimer. On USG Doppler, features of chronic venous thrombus like non-compressible venous segment, loss of phasic flow on the Valsalva maneuver, absent color flow if completely occlusive, lack of flow augmentation with calf squeeze, and increased flow in superficial veins were noted. Postoperatively, doppler findings of increased venous diameter, soft/deformable intraluminal material, smooth surface, and free-floating edge were considered features of acute thrombus.

The data was compiled and entered into an Excel sheet (Microsoft Corporation, Redmond, WA, USA) and analyzed for the prevalence of DVT in patients undergoing emergency laparotomies and the association of risk factors with its incidence. The APACHE II score was correlated with the occurrence of DVT. For statistical analysis, categorical variables were described as frequency and proportion, whereas continuous variables were described as mean ± standard deviation or median with an interquartile range as applicable. For inferential statistical analysis, the Chi-square or Fischer’s exact tests were used to compare proportions, whereas the student’s t-test and Mann-Whitney U test were used to compare means between two groups. Paired t-tests were used to compare the readings of continuous variables at two points in time.

## Results

Our study recruited 146 participants after applying inclusion and exclusion criteria and calculating the results. The age group in the study was 18-77 years (mean 43.55 ± 17); 50 participants (34.25%) were female, and 96 (65.75%) were male. The range for the BMI (kg/m2) was 14.52-30.06 (mean 20.77 ± 2.7). Among the 146 participants, various risk factors for DVT were recorded. Two patients (1.37%) were pregnant, 96 participants (65.75%) had a history of smoking, seven participants (4.79%) had disseminated abdominal malignancy, and 13 subjects (8.90%) had immunocompromised status. The duration of immobilization was 4-30 hours (mean 13.14 ± 5.78), and the duration of surgery was 1.5-6 hours (mean 3.4 ± 0.89). The most common cause of exploratory laparotomy was pre-pyloric/duodenal perforation (n=41, 28.08%), followed by ileal perforation (n=33, 22.60%). Among the acute intestinal obstructions, malignancy (n=16, 10.96%) was the most dominant cause, followed by band/adhesion (n=10, 6.85%) (Tables 1-2).

**Table 1:** Various causes of perforation peritonitis in the study group N: number of patients

Perforation peritonitis	N (%)
Pre-pyloric/duodenal perforation	41 (28.08)
Ileal perforation	33 (22.06)
Appendicular perforation	5 (3.42)
Colonic perforation	4 (2.74)
Caecal perforation	2 (1.37)
Rectal perforation	1 (0.68)
Gall bladder perforation	2 (1.37)
Cholecystectomy-duodenal fistula	2 (1.37)
Others	2 (1.37)

**Table 2:** Various causes of acute intestinal obstruction in the study group N: number of patients

Acute intestinal obstruction	N (%)
Malignancy	16 (10.96)
Stricture	6 (4.11)
Band/adhesion	10 (6.85)
Sigmoid volvulus	2 (1.37)
Bowel gangrene	6 (4.11)
Strangulated hernia	5 (3.42)
Gallstone ileus	1 (0.68)
Intussusception	2 (1.37)
Others	6 (4.11)

**Figure 1:**
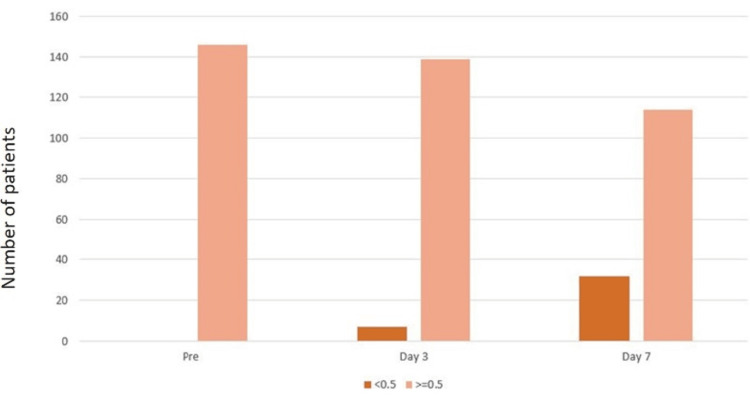
D-dimer value in the preoperative period and postoperative days 3 and 7

The D-dimer value was ≥0.5, i.e., positive, for all 146 patients for the preoperative period. On postoperative day 3, seven (4.8%) patients showed a D-dimer value of <0.5, and 32 (21.9%) patients showed a D-dimer value of <0.5 on day 7 (Figure 1).

**Figure 2:**
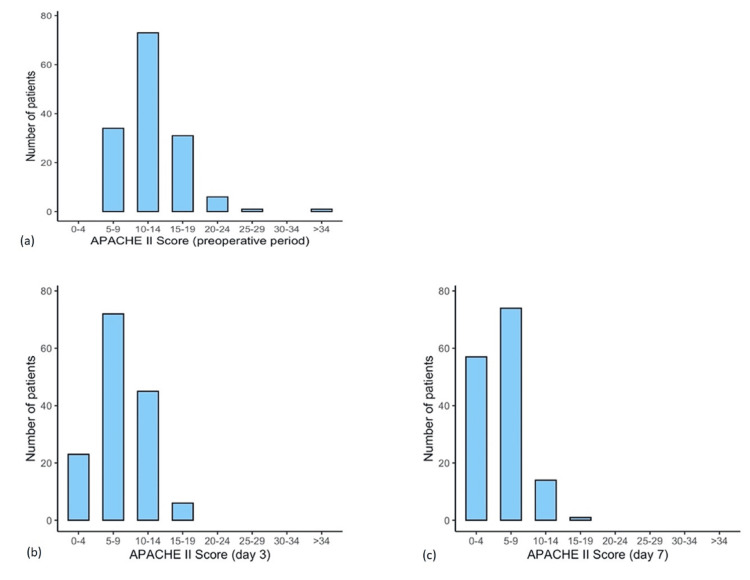
APACHE II score of patients in the preoperative period (a) and postoperative days 3 (b) and 7 (c)

**Figure 3:**
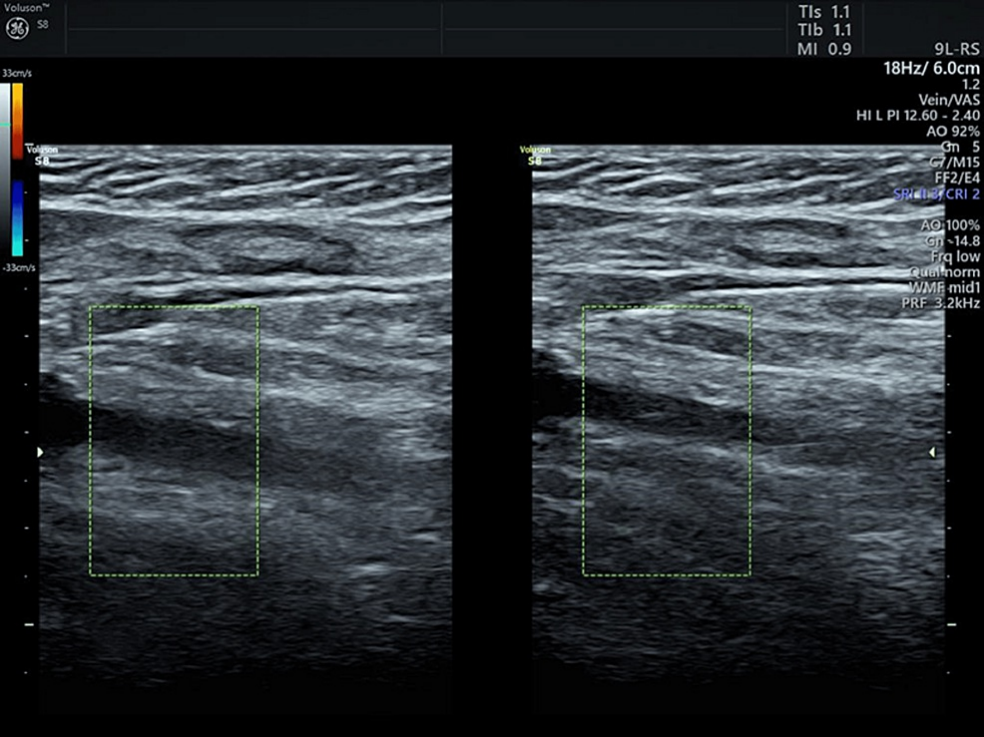
USG Doppler of the left lower limb showing a DVT in the superficial femoral vein, i.e., echogenic thrombus in distended vein and no color flow on Doppler USG: ultrasonography, DVT: deep venous thrombosis

The patients showed a pattern of decreasing APACHE II scores over time. For the preoperative period, no patient showed an APACHE II score between 0 and 4, whereas 23 (15.75%) patients and 57 (39.04%) patients had an APACHE II score between 0 and 4 on days 3 and 7, respectively. While two patients showed an APACHE II score of ≥25 for the preoperative period, no patient showed APACHE II scores of ≥25 on days 3 and 7 (Figure 2). In the preoperative period, USG Doppler was done to rule out DVT. On postoperative day 7, only one patient showed DVT in the USG Doppler (Figure 3).

## Discussion

The incidence of DVT varies depending on the type of surgery performed, with emergency exploratory laparotomies having a greater incidence of DVT than other procedures (Table 3). Out of 146 patients taken in our study, a single patient developed DVT in the postoperative period, i.e., 0.68% of emergency laparotomy patients. The patient who experienced DVT had no other known risk factors; however, his age of 60 years was a risk factor for DVT. Therefore, we may conclude that emergency laparotomy may be a separate risk factor for the emergence of DVT. One retrospective study by Vivekanand et al. reported that DVT and PE account for 1.28% of all complications in emergency laparotomy. However, being a retrospective study, preoperative DVT was not ruled out, unlike our prospective study [12].

**Table 3:** Previous studies investigating the prevalence of DVT in different sets of population EGS: emergency general surgery, DVT: deep venous thrombosis [15,11-17]

Study	Sample size	Study type	Study population	Postoperative DVT (%)
Literature from Western countries				
Amin et al., 2011 [14]	19,581	Retrospective observational	Major abdominal surgeries	1.6
Havens et al., 2015 [2]	24,068	Retrospective study	Acute Abdominal surgery	15.6
Gantz et al., 2020 [5]	-	Retrospective observational	EGS	0.623
Yang et al., 2020 [15]	767	Retrospective cohort	EGS	2.3
Ross et al., 2021 [16]	-	Retrospective cohort	EGS	1.9
Literature from Asian countries				
Liew et al., 2003 [11]	-	Retrospective review of published reports	All general surgeries	3-28
Vivekanand et al., 2015 [12]	100	Retrospective case series	Emergency exploratory laparotomy	1.28
Kuttanchettiyar et al., 2018 [13]	334	Retrospective descriptive study	Major abdominal surgeries	1.19
Chauhan et al,, 2017 [17]	400	Prospective observational study	All exploratory laparotomy	1 in 400

However, various studies have mentioned the role of a serum D-dimer cut-off value of 0.5mg/l to exclude the occurrence of DVT. Our study's findings are inconsistent because all the patients who underwent emergency exploratory laparotomies had elevated D-dimer readings (≥0.5 mg/l) before surgery. In the preoperative period, 100% (n=146) of the patients had D-dimer values that were ≥ 0.5, i.e., positive. So, based on the results of our study, we can say that the D-dimer test has limited significance in excluding DVT in preoperative patients undergoing emergency exploratory laparotomy before surgery. Thirty-two (21.9%) patients had a D-dimer value below 0.5 on postoperative day 7, and seven patients (4.8%) on postoperative day 3. As the number of postoperative days rises, there is a declining tendency in the value of D-dimer. The patient who developed DVT on postoperative day 7 had a quantitative D-dimer value of 2.8 mg/l, which falls in the group of 78.1% (n=114) who had raised D-dimer. Thus, based on the results of our investigation, just D-dimer is not useful for detecting DVT in the postoperative phase. However, the link between D-dimer readings and the occurrence of DVT could not be determined with significance in terms of p-value because we had only one patient with DVT.

The association between the postoperative APACHE II score and the development of DVT has not been investigated in any studies. Over time, the patients' APACHE II scores displayed a pattern of decline in our study. An APACHE II score of 14, 8, and 10 were recorded on preoperative, postoperative day 3, and postoperative day 7, respectively, for the patient later diagnosed with DVT. The association between the APACHE II score and the occurrence of DVT could not be deduced in any significant way from our investigation.

Limitations

Although we expanded the sample size to 146 (the calculated sample size was 106) with a 5% margin of error, a more extensive study might be conducted after reducing the margin of error. The sample size could have impacted the results. This study was done in the emergency surgery pool of a single tertiary healthcare institution. The spectrum of emergency surgeries can vary between healthcare institutions, so generalizing the results obtained is justified. Patients were followed for DVT until postoperative day 7, which limits the long-term sequelae.

## Conclusions

Our study's low prevalence (0.68%) of DVT in emergency exploratory laparotomy patients can be attributable to our institution's adherence to the ERAS protocol, a briefer follow-up period, and probably due to the exclusion of intubated patients, which includes a subset of high-risk sick patients with postoperative DVT. The D-dimer test plays a minimal role in excluding DVT in the preoperative and postoperative periods in these subsets of patients. USG Doppler can be used as a first investigation to rule out DVT. Future studies with larger sample sizes will be needed to determine the prevalence of DVT in patients undergoing emergency exploratory laparotomies.

## References

[1] Bevis PM, Smith FC (2016). Deep vein thrombosis. Surgery.

[2] Havens JM, Peetz AB, Do WS (2015). The excess morbidity and mortality of emergency general surgery. J Trauma Acute Care Surg.

[3] Cohen AT, Tapson VF, Bergmann JF (2008). Venous thromboembolism risk and prophylaxis in the acute hospital care setting (ENDORSE study): a multinational cross-sectional study. Lancet.

[4] McLendon K, Goyal A, Attia M (2023). Deep venous thrombosis risk factors. StatPearls [Internet].

[5] Gantz O, Mulles S, Zagadailov P, Merchant AM (2020). Incidence and cost of deep vein thrombosis in emergency general surgery over 15 years. J Surg Res.

[6] Khadilkar R, Bhatnagar SP, Dave P (2017). Causative factors of deep vein thrombosis of lower limb in Indian population. Int Surg J.

[7] Koperna T, Semmler D, Marian F (2001). Risk stratification in emergency surgical patients: is the APACHE II score a reliable marker of physiological impairment?. Arch Surg.

[8] Olaf M, Cooney R (2017). Deep venous thrombosis. Emerg Med Clin North Am.

[9] Lee H, Lim CW, Hong HP, Ju JW, Jeon YT, Hwang JW, Park HP (2015). Efficacy of the APACHE II score at ICU discharge in predicting post-ICU mortality and ICU readmission in critically ill surgical patients. Anaesth Intensive Care.

[10] Galanaud JP, Laroche JP, Righini M (2013). The history and historical treatments of deep vein thrombosis. J Thromb Haemost.

[11] Liew NC, Gul Y, Moissinac K (2003). Postoperative venous thromboembolism in Asia: a critical appraisal of its incidence. Asian J Surg.

[12] Vivekanand KH, Mohankumar K, Prakash D, Vikranth SN, Suresh TN (2015). Clinical outcome of emergency laparotomy: our experience at tertiary care centre (a case series). Int J Biomed Adv Res.

[13] Kuttanchettiyar KC, Chisthi MM (2018). Deep venous thrombosis after major abdominal surgeries: a tertiary level centre study. Int Surg J.

[14] Amin AN, Lin J, Thompson S, Wiederkehr D (2011). Inpatient and outpatient occurrence of deep vein thrombosis and pulmonary embolism and thromboprophylaxis following selected at-risk surgeries. Ann Pharmacother.

[15] Yang M, Murphy PB, Allen L, Sela N, Govind S, Leslie K, Vogt K (2020). Venous thromboembolism in emergency general surgery patients: a single-centre retrospective cohort study. Can J Surg.

[16] Ross SW, Kuhlenschmidt KM, Kubasiak JC (2020). Association of the risk of a venous thromboembolic event in emergency vs elective general surgery. JAMA Surg.

[17] Chauhan S, Chauhan B, Sharma H (2017). A comparative study of postoperative complications in emergency versus elective laparotomy at a tertiary care centre. Int Surg J.

